# Vitamin D and Cardiovascular Disease: Current Evidence and Future Perspectives

**DOI:** 10.3390/nu13103603

**Published:** 2021-10-14

**Authors:** Nicola Cosentino, Jeness Campodonico, Valentina Milazzo, Monica De Metrio, Marta Brambilla, Marina Camera, Giancarlo Marenzi

**Affiliations:** 1 Centro Cardiologico Monzino IRCCS, 20138 Milan, Italy; nicola.cosentino@ccfm.it (N.C.); jeness.campodonico@ccfm.it (J.C.); valentina.milazzo@ccfm.it (V.M.); monica.demetrio@ccfm.it (M.D.M.); marta.brambilla@ccfm.it (M.B.); marina.camera@ccfm.it (M.C.); 2 Department of Clinical Sciences and Community Health, University of Milan, 20122 Milan, Italy; 3 Department of Pharmaceutical Sciences, University of Milan, 20133 Milan, Italy

**Keywords:** vitamin D, cardiovascular disease, coronary artery disease, heart failure, atrial fibrillation, prevalence, prognosis

## Abstract

Vitamin D deficiency is a prevalent condition, occurring in about 30–50% of the population, observed across all ethnicities and among all age groups. Besides the established role of vitamin D in calcium homeostasis, its deficiency is emerging as a new risk factor for cardiovascular disease (CVD). In particular, several epidemiological and clinical studies have reported a close association between low vitamin D levels and major CVDs, such as coronary artery disease, heart failure, and atrial fibrillation. Moreover, in all these clinical settings, vitamin deficiency seems to predispose to increased morbidity, mortality, and recurrent cardiovascular events. Despite this growing evidence, interventional trials with supplementation of vitamin D in patients at risk of or with established CVD are still controversial. In this review, we aimed to summarize the currently available evidence supporting the link between vitamin D deficiency and major CVDs in terms of its prevalence, clinical relevance, prognostic impact, and potential therapeutic implications.

## 1. Introduction

Cardiovascular disease (CVD) is the main cause of morbidity and mortality in western countries, despite improvements in current preventive and therapeutic strategies [1]. In addition to the traditional and most recognized risk factors for CVD, new risk factors are emerging with potentially relevant prognostic and therapeutic implications [2]. Among them, hypovitaminosis D has been the focus of recent interest [3]. Low vitamin D levels are frequently observed across all ethnicities and among all age groups, occurring in 30–50% of the population [4,5]. Besides vitamin D’s established role in calcium homeostasis, its insufficiency or deficiency has been closely associated with diabetes mellitus, arterial hypertension, and chronic kidney disease [6,7]. Moreover, the detection of a nuclear vitamin D receptor (VDR) on vascular endothelial cells and cardiomyocytes has suggested a direct involvement of vitamin D in CVD development and progression, paving the way for a growing number of studies investigating this potential link [8,9,10]. To date, the cardiovascular clinical settings in which vitamin D has been widely investigated include coronary artery disease (CAD), heart failure (HF), and atrial fibrillation (AF), the three most frequent CVDs worldwide [1,3]. In particular, in all these conditions, vitamin D deficiency is a highly prevalent comorbidity and is associated with worse short-term and long-term prognosis [11,12]. According to this evidence, vitamin D supplementation has been proposed for the prevention and treatment of various CVDs, although research is still ongoing due to conflicting results [11,12].

In this review, we aimed to summarize the currently available evidence supporting the link between vitamin D deficiency and major CVDs in terms of its prevalence, clinical and prognostic relevance, and potential therapeutic implications. We also focused on current challenges and future perspectives in this field.

## 2. Vitamin D and the Cardiovascular System

**Figure 1:**
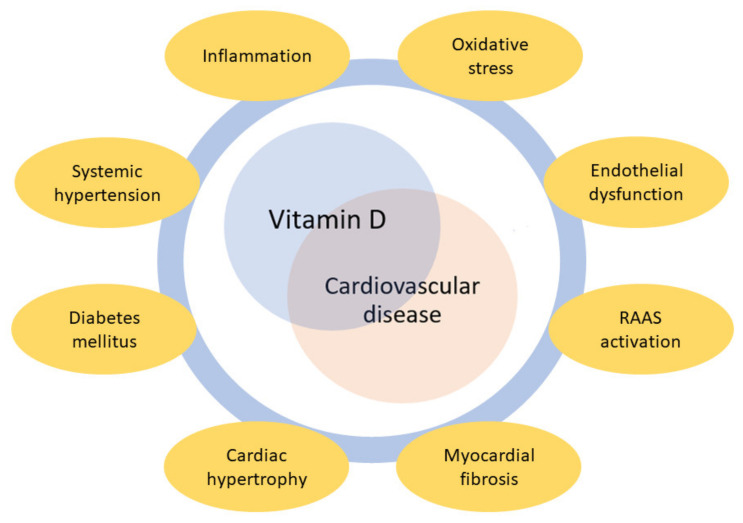
Major hypothetical mechanisms underlying the association between vitamin D and cardiovascular disease. RAAS = renin–angiotensin–aldosterone system.

A growing amount of data has highlighted the link between vitamin D and the cardiovascular system (Figure 1).

Cardiomyocytes, smooth muscle cells, fibroblasts, and vascular endothelial cells express VDR and 1α-hydroxylase, an enzyme that allows the production of the active form of vitamin D [13,14,15]. Experimental models have demonstrated that vitamin D has several cardiovascular effects, including anti-hypertrophic properties, inhibition of cardiomyocyte proliferation, stimulation of vascular smooth muscle cell proliferation, expression of vascular endothelial growth factor, and inhibition of both the renin–angiotensin–aldosterone system (RAAS) and natriuretic peptide secretion [16,17]. In particular, VDR activation by calcitriol or its analogues can directly inhibit angiotensin I expression and local angiotensin II generation in myocardium, renal arteries, and kidney tissue [18]. Moreover, vitamin D has been shown to induce angiotensin-converting enzyme 2 expression, the enzyme that cleaves angiotensin II to angiotensin 1–7, further counteracting the excess of angiotensin II and, hence, promoting the anti-fibrotic, anti-inflammatory, and anti-hypertensive functions of angiotensin 1–7 [19,20]. Lastly, immune cells that lack VDR have been reported to directly impact on the secretion of miR-106b-5p, which, in turn, may increase renin production by acting on juxtaglomerular cells, thus suggesting the role of inflammation as the cause of renin-driven hypertension [21].

Effects of vitamin D on myocytes’ contractility, by increasing intracellular calcium content, have been described [22]. Vitamin D also regulates the expression of certain metalloproteinases and inhibitors of metalloproteinases, influencing HF development [16,17,22]. In addition to its direct effects, vitamin D may indirectly affect the cardiovascular system through its influence on cardiovascular risk factors. Indeed, vitamin D deficiency has been associated with development of systemic hypertension [3,11,12], dyslipidemia, and type 2 diabetes mellitus [7,11]. Finally, emerging evidence suggests its anti-inflammatory role by inhibiting tumor necrosis factor alpha and nuclear factor kappa B activation and by stimulating interleukin 10, recognized key players in CVD [23,24].

Taken together, these mechanistic observations strongly support the involvement of vitamin D in the development and progression of CVD and its potential impact on short-term and long-term cardiovascular outcomes.

## 3. Vitamin D and Coronary Artery Disease

The association between vitamin D deficiency and CAD has been investigated in several studies [25,26,27]. In 1978, a Danish report initially found that vitamin D levels were significantly lower in patients with stable angina or acute myocardial infarction (AMI) than in controls [28]. In 1990, a case–control study confirmed that AMI patients had lower vitamin D levels than controls and, interestingly, the relative risk of AMI decreased across increasing quartiles of vitamin D [29]. These initial findings have also been reproduced in more contemporary cohorts (Table 1).

The association between low vitamin D levels and increased AMI risk seems to be maintained even after adjustment for major cardiovascular risk factors, as demonstrated in the large Health Professionals Follow-up Study [36]. In this study, 18,225 men were followed up for up to 10 years and those with vitamin D levels ≥30 ng/mL had approximately half the adjusted risk of AMI [36]. These data are in agreement with the results of a following meta-analysis showing an adjusted relative risk of 1.5 for cardiovascular events and of 1.4 for CAD, when comparing the lowest to the highest categories of vitamin D levels [37]. Low vitamin D levels seem to be associated not only with CAD risk but also with its severity. Indeed, in patients undergoing elective coronary angiography, vitamin D deficiency was associated with the presence of multi-vessel and more diffuse CAD and with more severe coronary artery stenosis [38,39]. Finally, VDR gene polymorphisms have been linked to stent restenosis following percutaneous coronary intervention [40]. Interestingly, vitamin D has been shown to strongly induce miR-145, the most abundant miRNA in vascular smooth muscle cells and the master regulator of their contractile phenotype [41]. In particular, decrease in miR-145 has been associated with all major deleterious changes in vascular health, including stenosis and vascular calcification [42].

Based on this evidence, the consistent finding of a high prevalence of vitamin D deficiency in patients hospitalized with AMI, ranging from 70% to 95% in different studies (Table 1), is not unexpected [30,31,43]. Of note, severe deficiency was observed in 10–40% of AMI patients [43]. Moreover, in this clinical setting, low vitamin D levels have been associated with worse short-term and long-term outcomes [30,31,32,43]. The first evidence of the independent link between vitamin D deficiency and increased in-hospital mortality in patients with AMI was provided by Correia et al. [33]. In their study, the authors reported that patients with vitamin D levels ≤10 ng/mL had a significantly higher in-hospital mortality than patients with vitamin D levels >10 ng/mL (24% vs. 5%) [33]. A similar association was reported in other studies (Table 1). Even more robust evidence has been provided on the long-term impact of low vitamin D levels on cardiovascular outcomes in AMI patients. Ng et al. [32] evaluated 1259 AMI patients and observed that the lowest vitamin D quartile (<7.3 ng/mL) was associated with long-term (median 1.5 years) re-hospitalization for acute HF and for recurrent AMI. Similarly, in the De Metrio et al. [34] study, the lowest quartile of vitamin D (<9 ng/mL) was a strong predictor of mortality and re-hospitalization for acute HF in 814 AMI patients followed-up for 1 year, with adjusted hazard ratios of 2.5 and 2.7, respectively. A hypothetical mechanism underlying the association between low vitamin D levels and worse long-term cardiovascular outcome in AMI patients has been recently suggested by Padoan et al. [44], who investigated the impact of admission vitamin D levels on left ventricular adverse remodeling in a prospective cohort of 253 patients. They confirmed that patients developing adverse remodeling after AMI had a lower median vitamin D level at the time of the index event and a higher risk of HF and mortality. Notably, no differences between patients with and without left ventricular adverse remodeling were found in terms of age, gender, cardiovascular risk factors, timing of coronary revascularization, cardiac function, or cardiovascular medications [44].

Despite these observations, there are no current conclusive results supporting the benefit of vitamin D supplementation as a strategy for cardiovascular protection in CAD [45,46,47]. On the one hand, data regarding vitamin D supplementation in primary prevention are sparse and controversial [35,48,49,50]. On the other hand, the potential benefit of vitamin D administration in the early phase of AMI has not been investigated yet. Finally, clinical trials specifically focusing on the benefit of vitamin D supplementation in AMI patients, in terms of long-term hard endpoints, are lacking and only a few studies are ongoing (ClinicalTrials.gov data) investigating its impact on surrogate primary endpoints, such as left ventricular remodeling and inflammation (Table 2).

## 4. Vitamin D and Heart Failure

Heart failure is a complex syndrome secondary to structural and/or functional cardiac abnormalities. Despite significant advances in therapeutic options over recent decades, it still represents a leading cause of mortality and morbidity worldwide [51]. Several mechanisms are involved in the pathogenesis of HF, including hemodynamic impairment, neurohormonal activation, enhanced inflammation, and micronutrients availability [51], that may explain the non-optimal impact of current therapies on clinical outcomes.

Several studies reported an association between HF and low vitamin D levels [52,53,54,55,56,57,58,59,60,61,62] (Table 3).

Initially, Shane et al. [53] found that about 25% of patients with advanced HF being evaluated for heart transplant had low serum vitamin D levels. Since then, a growing number of studies have reported lower vitamin D levels in patients with HF compared to a control group, ranging from 20% to 90% depending on the threshold used for the definition of vitamin D insufficiency and the investigated study population. The Intermountain Healthcare system study, including more than 40,000 subjects from a general population with at least one vitamin D measurement, found a progressive increase in HF prevalence as vitamin D levels decrease. Vitamin D levels also inversely correlated with the risk of developing HF at a mean 1.3-year follow-up, with an adjusted risk of HF of 2.0 and 1.3 for very low levels (≤15 ng/mL) and low levels (16–30 ng/mL), respectively [54]. Moreover, an independent and inverse correlation between vitamin D levels and N-terminal pro-brain natriuretic peptide (NT pro-BNP) levels and New York Heart Association (NYHA) functional class, as well as a direct correlation with left ventricular ejection fraction values, were observed in the Ludwigshafen Risk and Cardiovascular Health (LURIC) study [55]. Recently, low vitamin D levels have also been associated with a reduced functional capacity and with an increased rate of hospitalization in HF patients with preserved ejection fraction [56]. Thus, vitamin D insufficiency seems to be associated not only with higher HF prevalence and risk but also with its clinical severity and risk of hospitalization. In agreement with these observations, a higher early mortality risk has been reported in 10,974 patients hospitalized with HF and with low vitamin D levels [57]. In another study, progressively lower vitamin D levels were independently associated with higher HF hospitalization and all-cause mortality rates in 548 HF patients followed-up for 18 months [58]. Several other studies focusing on ambulatory or hospitalized HF patients confirmed the close association between vitamin D deficiency and worse prognosis (Table 3).

On the basis of these observations, the role of vitamin D supplementation in the prevention and treatment of HF has been investigated. Beneficial effects of vitamin D on left ventricular structure and function, reported in the Vitamin D Treating Patients with Chronic Hearth Failure (VINDICATE) study [63], and decreased RAAS activity observed with short-term vitamin D supplementation in chronic HF patients [63,65] highlight its potential therapeutic role in this clinical setting. In particular, the VINDICATE trial demonstrated that high-dose vitamin D supplementation for 1-year in chronic HF patients with vitamin D deficiency, given on top of contemporary optimal medical therapy, results in a significant improvement in left ventricular function [63]. However, interventional trials of vitamin D supplementation in patients at risk of developing HF and in those with chronic HF provided inconclusive results in terms of improvement in cardiovascular mortality and morbidity [64,66,67,68] (Table 3). In particular, vitamin D supplementation seemed to decrease serum levels of inflammatory markers and improve quality of life in patients with chronic HF, without reducing mortality or improving left ventricular function [67,68]. Several vitamin D supplementation regimens (such as high-dose and low-dose) and frequencies (daily, weekly, and monthly) for different durations (short-term and long-term) were used in clinical trials that enrolled heterogeneous study populations, possibly explaining the conflicting results [69]. Given the low cost and safety of vitamin D supplementation, the question of whether vitamin D benefits HF patients should still be tested in well-designed randomized trials. Such trials should focus on HF patients who would be most likely to benefit from vitamin D supplementation, in particular those with low vitamin D levels at baseline and high mortality risk.

## 5. Vitamin D and Atrial Fibrillation

Atrial fibrillation is the most common arrhythmia, affecting almost 2% of the general population, and it is a leading cause of stroke, death, and health care burden [70]. Despite identification of important risk factors for AF, such as old age, arterial hypertension, HF, CAD, valvular heart disease, surgery, and hyperthyroidism, a significant fraction of its risk remains currently unexplained [71]. Recent studies showed that low vitamin D levels are associated with AF and may be implicated in its pathogenesis, thus suggesting the potential of vitamin D to also become a therapeutic target in this clinical setting [72,73,74,75,76,77,78,79,80,81,82,83,84,85,86,87].

The potential mechanisms through which vitamin D may increase AF risk are either direct actions on the atrium or indirect modulation of cardiovascular risk factors. Low vitamin D levels are associated with inflammation which, in turn, has a critical role in the pathogenesis of AF [74]. Indeed, the risk of AF has been reported to be up to two-fold higher in patients with high C-reactive protein values [75]. Moreover, low vitamin D level might increase the AF risk due to RAAS activation, which influences structural and electrical atrial remodeling and regulates inflammatory responses [76]. Accordingly, low vitamin D levels are associated with more extensive left atrial fibrosis [77]. Finally, low vitamin D status may contribute to AF by increasing risks factors for its development, such as diabetes mellitus, hypertension, HF, and CAD [3].

Despite this evidence, clinical data on the relationship between vitamin D and AF are controversial (Table 4).

On the one hand, observational studies have shown that patients with vitamin D deficiency are about twice as likely to have AF as patients with normal vitamin D levels [72,79]. On the other hand, prospective studies have failed to find such an association [80,81]. Most studies evaluated the association between vitamin D and AF in the general population or in patients with CVD. However, in studies focusing on the incidence of post-operative AF, especially after coronary artery bypass graft (CABG) surgery, low vitamin D levels were consistently associated with a higher risk of AF occurrence (Table 4) [82,83,84]. In line with this, a recent dose–response meta-analysis found that vitamin D deficiency is moderately associated with an increased risk of AF in the general population and more robustly associated with post-operative AF in patients undergoing CABG. In particular, in this study, the relative risk of AF increased by 12% and 56% per each 10-unit decrease in vitamin D levels in the general population and in CABG patients, respectively, with evidence of a linear association [84]. Finally, Cerit et al. [85] found that vitamin D supplementation reduced post-operative AF in patients undergoing CABG and very low (<20 ng/mL) vitamin D levels. Moreover, vitamin D deficiency appears to be associated with a higher risk of AF in patients with chronic HF [86] and among younger but not older people [81]. Thus, further studies are needed to explore the population and the clinical setting where the association between low vitamin D levels and increased risk of AF is stronger and whether its supplementation can ultimately prevent AF.

## 6. Vitamin D and Interaction with Antiplatelet Drugs

Pharmacological therapy plays a crucial role in cardiovascular prevention and treatment. The link between low vitamin D levels and increased cardiovascular risk can also be explained by reduced drug efficacy or drug-resistance mechanisms documented in conditions of hypovitaminosis D. This aspect is particularly relevant for drugs that control platelet activation, an event that is deeply implicated in the pathogenesis of CAD.

Verdoia et al. [88] first evaluated the relationship between vitamin D status and platelet function in patients on dual antiplatelet therapy for the treatment of ACS or after percutaneous coronary intervention in stable CAD. The authors reported an inadequate inhibition of adenosine-di-phosphate (ADP)-mediated platelet aggregation by clopidogrel and ticagrelor in patients with lower vitamin D levels. More recently, Lu et al. [89] also showed that, in patients with ischemic stroke, clopidogrel resistance, defined according to the platelet maximum aggregation rate induced by ADP, is associated with significantly lower vitamin D levels than those measured in clopidogrel-sensitive patients. To date, the exact mechanism linking vitamin D to ADP-mediated platelet aggregation is not yet known. Experiments performed with VDR knock-out mice highlighted that the vitamin D/VDR system plays an important role in maintaining normal antithrombotic homeostasis in vivo [90]. Furthermore, VDR has also been localized in platelet mitochondria [91]. Therefore, one can assume vitamin D’s involvement in the regulation of platelet activation, which is a calcium-dependent event, through a nongenomic activity or direct modulation of calcium signaling [91,92]. This hypothesis is supported by the finding of a negative correlation between serum concentration of vitamin D and collagen-induced aggregation in individuals with normoglycemia, pre-diabetes, or diabetes mellitus who were not on aspirin [93]. Furthermore, ex vivo treatment with vitamin D of platelets from these cohorts of patients significantly reduced collagen- or ADP-induced platelet aggregation, thus supporting a positive role for vitamin D in inhibiting platelet aggregation [93]. Interestingly, a reduction in platelet reactivity—due to a significant increase in vitamin D levels—was observed in CAD patients under high-intensity statin treatment [94]. This finding, on the one hand, further supports the impact of vitamin D on platelet function and, on the other hand, provides an additional explanation of the “pleiotropic” benefits of statin therapy in CVD.

It is worth mentioning that vitamin D has an impact on intestinal and hepatic enzymes and transporters involved in drug metabolism and detoxification reactions [95,96,97]. This activity may also contribute to reduced drug efficacy during hypovitaminosis D, especially when enzymatic conversion of a prodrug into the active metabolite is needed. Compelling evidence indicates that vitamin D levels regulate CYP3A4 expression, the most important human drug-metabolizing enzyme with regard to a number of different drug substrates [97,98]. Verdoia et al. [94] recently addressed the impact of vitamin D on platelet reactivity and the rate of high-residual platelet reactivity (HRPR) in diabetic patients receiving dual antiplatelet therapy (aspirin and clopidogrel or ticagrelor or prasugrel) for ACS or elective percutaneous coronary intervention. They found a significant association between severe vitamin D deficiency and higher ADP-mediated platelet reactivity and HRPR, especially with the newer antiplatelet drugs ticagrelor and prasugrel. Although prasugrel is a pro-drug and CYP3A4 deficiency may account for a limited production of the active metabolite, the authors do not speculate on the association of hypovitaminosis D and ticagrelor, which does not require metabolism to inhibit platelet activation. Finally, given the close link between vitamin D levels and drug metabolism, it should be emphasized that seasonal changes in ultraviolet B and individual vitamin D levels might result in cyclic variation over the year in intestinal and hepatic clearance of therapeutic drugs [99]. However, despite these advances in understanding the pleiotropic effects of vitamin D, some gaps still need to be filled in order to identify the overall contribution of vitamin D to the inter-individual differences in drug metabolism and pharmacokinetics, and to assess whether vitamin D supplementation has the potential to influence drug metabolism and availability.

## 7. Future Perspectives

A close association between low vitamin D levels and CVD has been consistently reported; however, information on its causal link is still scarce. Epidemiological and interventional studies addressing the question of whether assessment of vitamin D offers additional information over traditional risk factors provided conflicting results. In particular, Mendelian randomization studies failed to demonstrate an association between genetic variants linked to vitamin D concentration and cardiovascular mortality, suggesting that low vitamin D levels are associated with, but unlikely to be causal for, higher cardiovascular risk [100]. Many reasons, related to both vitamin D supplementation and study design, may explain these conflicting results and should be taken into account when negative interventional trials are considered in patients with CVD. Differences in vitamin D supplementation, such as doses, baseline concentration, therapy duration, absorption and metabolism, definitions of vitamin D insufficiency/deficiency and differences in study population characteristics, inappropriate follow-up time, and lack of a true control group with normal vitamin D levels are the most important explanations. In particular, reviewing interventional trials focusing on cardiovascular patients analytically, two main common features that might explain their negative results can be identified. First, most of them involved patients generically at low cardiovascular risk. For example, the recent large randomized Vitamin D and OmegA-3 Trial (VITAL) enrolled 25,000 healthy men and women and excluded those with known cardiovascular disease [101]. Second, all vitamin D levels were considered at patients’ enrollment and also in the active arm of the interventional trials. These two possible limitations may warrant a re-examination of a central criterion of pragmatism—eligibility—in the outline of forthcoming cardiovascular trials investigating the role of vitamin D supplementation. Possibly, the ideal subset of patients for testing vitamin D administration might be represented by patients at high cardiovascular risk and with severe vitamin D insufficiency. Based on these considerations, additional investigation from ongoing and future randomized studies is needed to assess whether supplementation therapy with vitamin D has a role in the prevention and/or management of CVD. Finally, it should be taken into account that vitamin D status is greatly affected by lifestyle, diet, comorbidities, and seasonal variation, so a single snapshot of vitamin D measurement can hardly help to fully clarify the role of vitamin D in long-term cardiovascular risk. Therefore, other biomarkers, such as 1,25-dihydroxyvitamin D and 24,25-dihydroxyvitamin D, that more reliably reflect vitamin D status [102] should be investigated and validated as cardiovascular risk factors and then considered to potentially select patients who may benefit most from vitamin D supplementation.

## 8. Conclusions

In conclusion, the worsening worldwide trend toward insufficiency and the growing knowledge of its cardiovascular actions have sparked increasing interest in vitamin D in the prevention and treatment of major CVDs. Although epidemiological studies have reported a close association between low vitamin D levels and high cardiovascular risk, controversies still continue regarding the exact role of vitamin D in CVD and whether it represents a bystander-only indicator or a causal culprit that is amenable to treatment. Similarly, whether supplementation in patients at high cardiovascular risk and with vitamin D insufficiency can be of benefit in terms of improvement in outcomes remains uncertain and warrants further well-designed investigation.

## Figures and Tables

**Table 1:** Some of the major studies on the clinical relevance of vitamin D levels in patients with coronary artery disease.

Author	Country	Study Design/Type of Observation	Study Population	Patients (*n*) Age (yrs) Gender	Definition of Vit. D Deficiency (ng/mL)	Prevalence of Vit. D Deficiency	Major Findings
Raslan [26]	Egypt	Case–-control /prospective	CCS	*n* = 84	-	-	Every 10-unit (ng/mL) increase in vitamin D level decreased the chance of having chronic stable angina by three times
Xu [27]	China	Case–control /prospective	Postmenopausal women	*n* = 93 67 ± 6 yrs	Deficiency: <10	-	Vitamin D deficiency increased CAD risk (OR = 2.9)
Lee [30]	US	Retrospective	NSTEMI/STEMI	*n* = 219 58 ± 11 yrs 74% males	Insufficiency: 21–29 Deficiency: <20	75% insufficient; 21% deficient	-
Brøndum-Jacobsen [31]	Denmark	Population-based study /retrospective	General population	*n* = 10,170 57 yrs 44% males	-	-	Patients with low vitamin D levels had increased adjusted risks of CAD of 72%, of AMI of 99%, and of fatal CAD/AMI of 122% compared to those with higher vitamin D levels at 29-years FU
Ng [32]	England	Cohort study /prospective	NSTEMI/STEMI	*n* = 1259 66 ± 13 yrs 72% males	Deficiency: <20	74% deficient	A significant higher incidence of MACE in patients with vitamin D deficiency at 1.6-years FU
Correia [33]	Brazil	Retrospective	ACS	*n* = 20670 ± 12 yrs 50% males	Severe deficiency: <10	10% severely deficient	A significant higher incidence of in-hospital and long-term mortality (2-year FU) in patients with severe vitamin D deficiency
De Metrio [34]	Italy	Cohort study /prospective	NSTEMI/STEMI	*n* = 814 67 ± 12 yrs 72% males	Insufficiency: 21–29 Deficiency: <20	19% insufficient; 70% deficient	A higher incidence of in-hospital mortality, mechanical ventilation, and major bleeding in patients with the lowest quartile of vitamin D. A significant higher incidence of 1-year mortality and re-hospitalization for HF in patients with the lowest quartile of vitamin D
Scragg [35]	Mostly from New Zeland	RCT /prospective	Participants from the community; adults aged 50–84 years	*n* = 5108 66 ± 8 yrs 58% males	Oral vitamin D_3_ in an initial dose of 20,000 IU, followed a month later by monthly doses of 100,000 IU, or placebo for a median of 3.3 years		The primary outcome of CVD incidence and death occurred in 11.8% in the vitamin D group and in 11.5% in the placebo group (adjusted HR: 1.02 (95% CI, 0.87–1.20)). Similar results were seen for participants with baseline vitamin D deficiency and for secondary outcomes (angina, HF, hypertension, arrhythmias, arteriosclerosis, stroke, and venous thrombosis)

Legend: ACS = acute coronary syndrome; CCS = chronic coronary syndrome; CI = confidence interval; CVD = cardiovascular disease; FU = follow-up; HF = heart failure; HR = hazard ratio; IU = international unit; MACE = major adverse cardiac events; NSTEMI = non-ST-elevation myocardial infarction; OR = odds ratio; RCT = randomized controlled trial; STEMI = ST-elevation myocardial infarction; US = United States.

**Table 2:** Main current studies (recruiting or not yet recruiting) focusing on vitamin D clinical relevance or supplementation in patients with cardiovascular disease (ClinicalTrials.gov data, updated to 23 September 2021).

	Study #1	Study #2	Study #3	Study #4
ClinicalTrials.gov Identifier	NCT03416361	NCT02996721	NCT03086746	NCT02178410
Study start	December 2021	April 2017	April 2018	October 2012
Study type	Interventional	Interventional	Observational	Interventional
Study design	RCT	RCT	Prospective	RCT
Status	Not yet recruiting	Recruiting	Recruiting	Recruiting
Clinical setting	Stable HF	AMI	STEMI	General population
Interventions	4000 IU vitamin D3/day	Supplementation to maintain	2000 IU vitamin D/day or	Vitamin D levels > 40 ng/mL
	1 gr omega-3/day			
Main outcomes	2-year mortality and HF	2-year death, AMI	6-month 5% reduction in LVEF	7-year AF
	Hospitalization		HF hospitalization, CVA	15% increase in LVESVi
Estimated enrollment (*n*)	1253	890	800	25,871

Legend: AF = atrial fibrillation; AMI = acute myocardial infarction; CVA = cerebral vascular accident; HF = heart failure; IU = international unit; LVEF = left ventricular ejection fraction; LVESVi= left ventricular end-systolic volume indexed; RCT = randomized clinical trial; STEMI = ST-elevation myocardial infarction. **Study #1:** Vitamin D Treating Chronic Heart Failure (the Effect of Vitamin D Supplementation in Patients with Heart Failure) (VINDICATE2). **Study #2:** A Trial Evaluating Vitamin D Normalization on Major Adverse Cardiovascular-Related Events Among Myocardial Infarction Patients (TARGET-D). **Study #3:** Vitamin D as a Novel Determinant of Injurious Cardiac Remodelling After Acute Myocardial Infarction (VINDICATE-MI). **Study #4:** Vitamin D and OmegA-3 Trial (VITAL) Rhythm Study.

**Table 3:** Some of the major studies on the clinical relevance of vitamin D levels in patients with heart failure.

Author [Ref]	Country	Study Design/Type of Observation	Study Population	Patients (*n*) Age (yrs) Gender (Male)	Prevalence of Vitamin D Deficiency and Major Findings
Zitterman [52]	Germany	Case–control /prospective	NYHA ≥II	*n* = 53 60% males	Significant reduction of vitamin D levels in HF patients
Shane [53]	US	Cohort study /prospective	NYHA III–IV	*n* = 101 79% males	17% of the study patients had vitamin D levels <9 pg/mL
Anderson [54]	US	Cohort study /prospective	General healthcare population	*n* = 41,504	97% of HF patients had vitamin D levels <30 ng/mL 1.31 HR for new HF development in patients with low and very low vitamin D
Liu [58]	Netherlands	Cohort study /prospective	NYHA II–IV	*n* = 548 74 years 61% males	1.09 HR (95% CI 1.00–1.16) for all-cause mortality and re-hospitalization for HF at 18 months per 10 nmol/L decrease of vitamin D
Kim [59]	US	Retrospective	NHANES from 2001–2004	*n* = 8351	89% of HF patients with CAD had vitamin D <30 ng/mL
Gotsman [61]	Israel	Case–control/retrospective	Age ≥45 years	*n* = 49,834 49% males	1.52 HR (95% CI 1.21–1.92) for 1.6-year mortality in patients with vitamin D deficiency (<25 nmol/L). 0.68 HR (95% CI 0.54–0.85) for 1.6-year mortality in patients in therapy with vitamin D supplementation
Gruson [62]	Belgium	Cohort study /prospective	LVEF ≥35%	*n* = 1709 69 years 79% males	Vitamin D significantly predicted CV death and heart transplantation at 4.1 years
Witte [63]	Caucasian Non-Caucasian	RCT /prospective	LVEF ≤ 45% + NYHA II–III + vitamin D < 20 ng/mL	*n* = 229 69 ± 13 yrs 79% males	1-year of high-dose vitamin D supplementation (4000 IU daily) did not improve 6-min walk distance at 1 year but was associated with a significant improvement in LVEF and a reversal of LV remodeling
Zitterman [64]	Germany	RCT /prospective	NYHA ≥ II + vit D ≤ 75 nmol/L	*n* = 40,054 55 years 83% males	3-year mortality was not different in patients receiving vitamin D (4000 IU/day) or placebo with a HR of 1.09 (95% CI 0.69–1.71). Other secondary clinical endpoints were similar between groups (HF hospitalization, resuscitation, highly urgent listing for heart transplantation, heart transplantation, and hypercalcemia).

Legend: CHF = chronic heart failure; CI = confidence interval; CV = cardiovascular; HF = heart failure; HR = hazard ratio; LV = left ventricular, LVEF = left ventricular ejection fraction; NHANES = National Health and Nutrition Examination Survey; NYHA = New York Heart Association; RCT = randomized controlled trial.

**Table 4:** Some of the major studies on the clinical relevance of vitamin D levels in atrial fibrillation.

Author [Ref.]	Country	Study Design/Type of Observation	Study Population	Patients (*n*)	Age (yrs)	Gender (males)	Endpoint Considered	Major Findings
Chen [79]	China	Case–control /retrospective	-	322	65 ± 5	45%	Incidence of AF	Vitamin D <20 ng/mL increased AF risk by two times
Gode [83]	Turkey	Case–control /prospective	CABG	90	59 ± 6	33%	New-onset post-op AF	Vitamin D levels in AF patients: 9 ng/mL Vitamin D levels in non-AF patients: 15 ng/mL
Cerit [85]	Cyprus	Retrospective	On-pump CABG	128	65 ± 9	86%	New-onset post-op AF	Vitamin D levels in AF patients: 20 ng/mL Vitamin D levels in non-AF patients: 26 ng/mL
Ozcan [87]	Turkey	Case–control /prospective	Hypertensive patients	227	-	-	new-onset AF	Vitamin D <20 ng/mL increased AF risk by 1.7 times

Legend: AF = atrial fibrillation; CABG = coronary artery disease graft; post-op = post-operative.

## Data Availability

Not applicable.
